# Characteristics of an Iron-Reducing, Moderately Acidophilic Actinobacterium Isolated from Pyritic Mine Waste, and Its Potential Role in Mitigating Mineral Dissolution in Mineral Tailings Deposits

**DOI:** 10.3390/microorganisms8070990

**Published:** 2020-07-02

**Authors:** Ivan Nancucheo, D. Barrie Johnson

**Affiliations:** 1 Facultad de Ingeniería y Tecnología, Universidad San Sebastián, Lientur 1457, Concepción 4080871, Chile; 2 School of Natural Sciences, Bangor University, Deiniol Road, Bangor LL57 4UF, UK; d.b.johnson@bangor.ac.uk

**Keywords:** acidophile, *Curtobacterium ammoniigenes*, heterotroph, mine tailings, iron reduction

## Abstract

Reactive pyritic mine tailings can be populated by chemolithotrophic prokaryotes that enhance the solubilities of many metals, though iron-reducing heterotrophic microorganisms can inhibit the environmental risk posed by tailings by promoting processes that are the reverse of those carried out by pyrite-oxidising autotrophic bacteria. A strain (IT2) of *Curtobacterium ammoniigenes*, a bacterium not previously identified as being associated with acidic mine wastes, was isolated from pyritic mine tailings and partially characterized. Strain IT2 was able to reduce ferric iron under anaerobic conditions, but was not found to catalyse the oxidation of ferrous iron or elemental (zero-valent) sulfur, and was an obligate heterotrophic. It metabolized monosaccharides and required small amounts of yeast extract for growth. Isolate IT2 is a mesophilic bacterium, with a temperature growth optimum of 30 °C and is moderately acidophilic, growing optimally at pH 4.0 and between pH 2.7 and 5.0. The isolate tolerated elevated concentrations of many transition metals, and was able to grow in the cell-free spent medium of the acidophilic autotroph *Acidithiobacillus ferrooxidans*, supporting the hypothesis that it can proliferate in acidic mine tailings. Its potential role in mitigating the production of acidic, metal-rich drainage waters from mine wastes is discussed.

## 1. Introduction

Waste materials from metal mining, such as mineral tailings, have little or no economic value, making their exploitation not profitable. In the context of mine management, tailings have the potential to pose a long-term threat to the environment. Mine tailings are one of the major waste products generated during the mining of metal ores, and have variable physical and chemical compositions, dependent on the ore body being processed and the mining operations [1]. Following the crushing and grinding of ores (comminution), target minerals are segregated from other (gangue) minerals by froth flotation. The fine-grain mineral wastes produced (tailings) may account for up to 99% of the primary ore body [2]. While the mineralogical composition of tailings is highly variable, they frequently contain significant amounts of potentially acid-generating minerals, such as pyrite (FeS**_2_**), though the acidity generated in fresh tailings can be neutralized by basic materials, such as lime (CaO), that are often added to enhance froth flotation [3].

The dissolution of sulfide minerals requires water and an oxidizing agent, which may be either molecular oxygen or ferric iron, and may occur in either aerobic or anaerobic (micro) environments via mechanisms that have been widely reported [4]. In many cases, the potential for acid generation greatly exceeds the neutralization potential of tailings, and liquors within and draining from tailing deposits can become highly acidic, and enriched with soluble transition metals derived from the dissolution of residual sulfidic (e.g., chalcopyrite; CuFeS**_2_**) and other minerals. In addition, such waters are highly toxic to most life-forms [5]. The reactivity of pyritic mine tailings derives from their small particle size, and their content of acid-generating and metal-rich sulfidic minerals [6]. There have been a number of studies on the microbiology of tailing deposits located in different parts of the world [7,8,9,10]. Indigenous prokaryotes include well-known chemolitho-autotrophic acidophiles, such as *Acidithiobacillus* and *Leptospirillum* spp., and chemolitho-heterotrophic species (e.g., *Ferrimicrobium* and *Ferroplasma* spp.), which use the ferrous iron and/or reduced sulfur as electron donors. Most species of iron-oxidizing acidophiles that oxidize iron when oxygen is present can also use ferric iron as an alternative electron acceptor in anaerobic environments [11]. Some species of obligately heterotrophic acidophiles, including *Acidiphilium, Acidocella* and *Acidobacterium* which are able to reduce ferric iron but not oxidize ferrous iron, have also been identified in mine tailings [8]. Interestingly, heterotrophic acidophilic bacteria that reduce iron attach to sulfide minerals and form biofilms. Pyrite particles colonized with *Acidiphilium* and *Acidocella* spp. were found to be less susceptible to accelerated oxidation by mineral-oxidizing acidophiles, and a technique based on this observation, referred to as “bioshrouding”, was suggested as a method of partially securing reactive mine wastes [12].

In a series of mesocosm experiments, set up to examine how engineering the microbial communities of reactive mine tailings could be used to limit the generation of acidity and the release of metals [13], it was found that those that had either not been inoculated, or had been inoculated only with a mixed culture of iron-oxidizing chemolithotrophic acidophiles, became heavily colonized (~ 3 × 10^6^ colony forming units/g), within 12 months, by a bacterium that was identified (from its partial 16S rRNA gene sequence) as a strain of *Curtobacterium ammoniigenes* (99% gene similarity). This bacterium (and two others that were also isolated from the tailings) was inferred to have originated from the tailings themselves, and had not been completely eradicated by pre-treatment of the tailings, which were washed with strong (3 M) sulfuric acid to remove the residual lime. *C. ammoniigenes* is a heterotrophic, ammonium-oxidizing actinobacterium, the type strain of that had been isolated from water weeds growing in highly acidic (pH 2–4) swamps adjacent to acid sulfate soils in Vietnam [14]. There have been no previous reports of this bacterium in acidic mine-impacted environments, such as acid mine drainage, biomining sites, or waste rock and tailings deposits. *Curtobacterium ammoniigenes* strain IT2 has been shown to be a moderate-acidophile and an obligate heterotroph, which tolerates elevated concentrations of many transition metals, and also catalyses the dissimilatory reduction of ferric iron. These characteristics infer that is has a potential role in mitigating the formation and migration of acidic, metal-rich waters from tailings dumps.

## 2. Materials and Methods

### 2.1. Isolation and Cultivation of C. ammoniigenes *IT2*

The bacterium was originally isolated from pyritic tailing obtained from the Agua Blanca nickel–copper mine, Spain [13]. Briefly, homogenized tailing samples were serially diluted onto solid medium that contained 5 mM fructose/0.02% (*w*/*v*) yeast extract, acidophile basal salts (ABS) and trace elements (TE) [15], adjusted to pH 3.5 with sulfuric acid, and incubated aerobically for 14 days at 30 °C. The isolate was purified by repeated single-colony using the same medium and incubation conditions. After the purity of the isolate was confirmed, DNA was extracted and 16S rRNA genes were amplified, sequenced and compared to those in public databases, as described previously [16]. Single colony was transferred into liquid medium containing 5 mM/fructose/0.005% (*w*/*v*) yeast extract, and ABS adjusted to pH 3.5, incubated at 30 °C and shaken at 100 rpm.

### 2.2. pH and Temperature Characteristics

*C. ammoniigenes* IT2 was grown in batch mode in a bioreactor (1 L liquid volume in a 2 L reactor vessel; Electrolab Ltd., Tewkesbury, UK) under conditions of fixed temperature and pH. The liquid medium used contained 5 mM fructose/0.005% (*w*/*v*) yeast extract, and ABS/TE and cultures were aerated at 1 L/min and stirred at 150 rpm. To determine the effect of pH on its growth, cultures were maintained at 30 °C and varying pH (2.7–5.0; controlled by automated addition of 0.1 M NaOH), and to determine the effect of temperature, cultures were maintained at between 22 and 37 °C with pH maintained at 4.0. Samples were withdrawn from the reactor at regular intervals, the optical densities at 600 nm were measured (OD_600_), and culture doubling times were evaluated from semi-logarithmic plots of changes in OD_600_ against time.

### 2.3. Organic Nutrition of C. ammoniigenes *IT2*

The ability of the isolate IT2 to grow in the absence of yeast extract was tested by comparing growth in 5 mM fructose/ABS/TE liquid medium with and without yeast extract. The isolate was also tested for growth in liquid medium (pH 4.0, containing 0.005% yeast extract and ABS/TE) amended with various organic compounds, including sugars, alcohols, aliphatic acids and amino acids (listed in Table 1). Different concentrations of substrates were used to approximately equalize their carbon-equivalents (i.e., 5 mM for C_6_ substrates, 10 mM for C_3_ substrates, etc.). Replicate universal bottles were incubated 30 °C and OD_600_ were measured over 7 days.

### 2.4. Oxidation of Iron and Reduced Sulfur

*C. ammoniigenes* IT2 was assessed for its ability to grow autotrophically in oxic medium, using ferrous iron or elemental sulfur as electron donors in organic carbon-free medium, containing 5 mM ferrous sulfate (pH 3.0) or 1% (*w*/*v*) elemental sulfur (pH 3.5). Non-inoculated controls were also prepared. The ability to oxidize ferrous iron and sulfur when grown heterotrophically was also tested in the same medium, amended with 0.002% (*w*/*v*) yeast extract. Oxidation of ferrous iron was determined by measuring changes in concentrations of ferrous iron using the ferrozine assay [17], and changes in culture pH as a measure of sulfur oxidation, again relative to non-inoculated controls.

### 2.5. Growth of C. ammoniigenes *IT2* in Spent Medium of Acidithiobacillus ferrooxidans

*Acidithiobacillus (A.) ferrooxidans* (ATCC 23270^T^) was grown in batch mode in a bioreactor using 5% (*w*/*v*) elemental sulfur as electron donor and ABS/TE, at pH 1.8 and 30 °C. The bioreactor was stirred at 150 rpm and aerated at 1 L/min. After 25 days of growth, the culture was removed and cell-free culture liquors was obtained by centrifugation (10,000× *g*; 15 min) followed by filtration through 0.2 µm (pore size) cellulose nitrate membrane filters (Whatman, UK). Cell-free culture liquors were adjusted to pH 3.5 with 1 M NaOH, filtered, and 30 mL aliquots were dispensed into 100 mL conical flasks. These were then inoculated, in duplicate, with *C. ammoniigenes* IT2, and a third (non-inoculated) duplicate set used as sterile controls. Cultures were incubated and shaken at 150 rpm, at 30 °C for up 12 days, and samples were withdrawn at days 0, 4, 8 and 12 to enumerate bacterial cells (using a Thoma counting chamber and Leitz phase contrast microscope). Concentrations of glycolic acid were determined by a colorimetric technique [18], and total dissolved organic carbon (DOC) was measured using a LABTOC DOC analyzer (Pollution and Process Monitoring, UK).

### 2.6. Tolerance of C. ammoniigenes *IT2* to Some Transition Metals

To evaluate its tolerance to four transition metals frequently often found in elevated concentrations in pyritic tailings, *C. ammoniigenes* IT2 was grown in liquid medium containing 5 mM fructose, 0.005% (*w*/*v*) yeast extract, ABS and TE, supplemented with sterile solutions of copper, ferrous, nickel and zinc sulfates (concentrations used listed in Table 2). Culture pH was adjusted to pH 4.0 with sulfuric acid, and pH 2.7 in the case of ferrous iron (to minimize chemical oxidation and precipitation of iron), and the cultures were incubated at 30 °C for 7 days. Positive growth was assessed from OD_600_ measurements. In the case of ferrous iron, where culture turbidity was also due to some abiotic formation of ferric iron, growth was assessed by measuring changes in fructose concentrations using ion chromatography [19], and concentrations of ferrous iron were also measured after 7 days to determine the amount of this metal remaining in the solution.

## 3. Results and Discussion

Pale yellow colonies of strain IT2 were observed, after 7 days of incubation, on yeast extract/fructose solid medium pH 3.5. Cells were non-motile rods of irregular shape, and did not appear to produce endospores. The isolate did not oxidize elemental sulfur or ferrous iron autotrophically or heterotrophically. It grew poorly on defined single carbon sources, but the addition of small amounts of yeast extract promoted growth on sugars, fructose in particular, presumably due to the requirement of one or more growth factors (Table 1). The isolate was able to use a relatively limited range of defined organic substrates, including glucose, fructose and galactose, compared with *Acidobacterium capsulatum* and *Acidiphilium cryptum* also found in mine tailings. Its substrate range differed from other acidophilic bacteria found in mine tailings [20]. All of the amino acids tested inhibited the growth of the bacterium at the concentrations tested (i.e., growth was less than in the presence of yeast extract alone), even though they grew well on tryptone soya broth. It was also noted that some of the low molecular weight organic acids tested, such as acetic and citric acid, inhibited the growth of strain IT2 [21]. The absence of growth in an organic carbon-free medium suggested that *C. ammoniigenes* IT2 is an obligate heterotroph.

**Figure 1:**
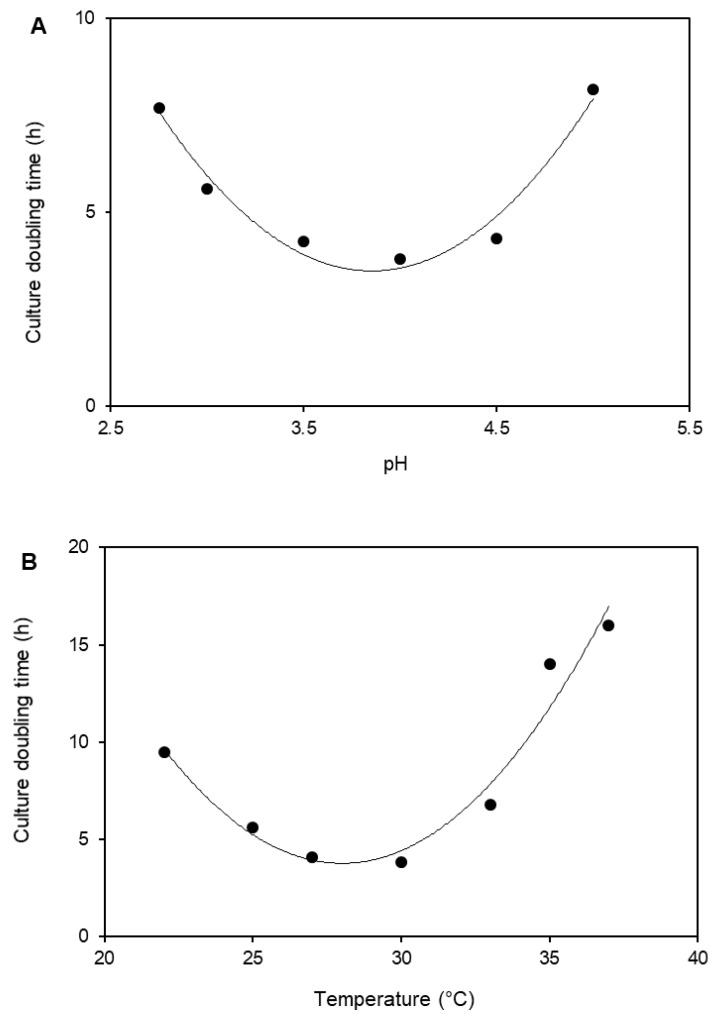
Effect of (**A**) pH (at 30 °C) and (**B**) temperature (at constant pH of 4.0) on the growth of isolate IT2.

Culture doubling times of isolate IT2 grown at different temperatures and pH values are shown in Figure 1. Strain IT2 grew between pH 2.7 and 5.0, with a pH growth optimum of 4.0. The optimum temperature for growth was found to be at 30 °C, and the maximum temperature at which growth was observed at 37 °C. Under optimum conditions of temperature and pH, its culture doubling time was 3.8 h, equivalent to a growth rate of 0.18 h^−1^. The isolate was unable to grow below pH 2.7 and above pH 5.0, indicating that it is a moderate acidophile. Strain IT2 is therefore more tolerant of extreme acidity than *C. ammoniigenes* B55^T^ (pH range 3.5–8.0, with optimal growth at pH 4.0). All other species of *Curtobacterium* are neutrophiles [14].

In addition, *Curtobacterium* isolate IT2 showed similar copper tolerance to that reported for *Acidiphilum cryptum* [22], a heterotrophic acidophilic bacteria that has also been found in pyritic mine tailings [8] and can also grow on a wide range of monosaccharides, dicarboxylic acids and tricarboxylic acids. As is the case with many other acidophilic bacteria, isolate IT2 exhibited a high tolerance to elevated concentrations of ferrous iron, zinc and nickel (Table 2), which helps to explain why it is able to proliferate in pyritic tailings [13]. Tolerance to transition metals is another major characteristic of heterotrophic bacteria isolated from mineral tailings, though, in general, heterotrophs are less tolerant to dissolved metals than iron-oxidizing chemolithotrophs (*A*. *ferrooxidans* in particular) [23].

**Figure 2:**
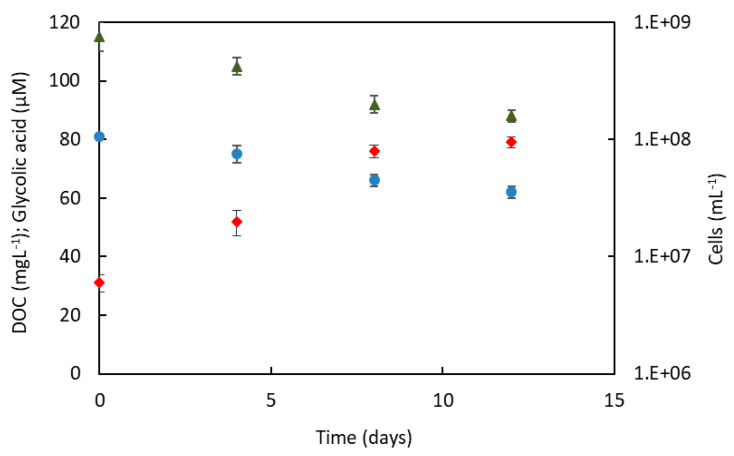
Growth of *C. ammoniigenes* IT2 on spent *A. ferrooxidans* medium. (♦) number of cells; (●) dissolved organic carbon; (▲); glycolic acid. The symbols indicate means for duplicate cultures, and error bars indicate range.

*C. ammoniigenes* IT2 was able to grow in the spent medium of *A*. *ferrooxidans*. Figure 2 shows that numbers of *C. ammoniigenes* cells increased by over one order of magnitude within 12 days, and that this was accompanied by a decrease in the concentration of total DOC. However, only about 24% of the total DOC was metabolized over this period, and the cessation of growth of *C. ammoniigenes* isolate suggests that the residual DOC was not metabolized by this strain. No changes in the concentration of DOC were observed in the control cultures containing sterile spent medium of *A. ferrooxidans,* which were also confirmed to be devoid of bacterial cells (data not shown). This result provides further support that primary-producing chemolitho-autotrophic acidophilic bacteria, such as *A*. *ferrooxidans,* can support the growth of heterotrophic bacteria by providing them with electron donors and carbon sources. Diaby et al. [8] proposed a model using the microbiological and geochemical results to explain how autotrophic acidophiles sustained the growth of heterotrophic iron-reducers present in mine tailings at the Andina mine, CODELCO, Chile. DOC, mainly lysates and exudates from *A. ferrooxidans* and other primary producers, was proposed to sustain the heterotroph communities dominated by *Acidiphilium*, *Acidocella* and *Acidobacterium* spp. Carbon transfer between acidophilic prokaryotes that either fix or produce CO_2_ was demonstrated by Kermer et al. [24], using protein-based stable isotope probing, to be a two-way process. The syntrophic relationship of the acidophilic species involves organic carbon, derived from autotroph (as exudates or cell lysates), being used as the carbon source by heterotrophic bacteria, and CO**_2_** generated by heterotrophic species being using as carbon source by autotrophs. The latter is particularly important in low pH environments, where the solubility of CO**_2_** is very low [25]. Previously, Schnaitman and Lundgren [26] had shown that 10% of labelled carbon (**^14^**CO_2_) was leaked by *A. ferrooxidans* into its growth medium, and pyruvic acid was identified as one the low molecular weight exudates. Besides, low molecular weight carboxylic acids, such as formate, acetate and pyruvate, were detected from two copper mine tailings [27].

Nancucheo and Johnson [28] reported that glycolic acid was produced and excreted by mineral-oxidizing bacteria, such as *L. ferriphilum*, *Acidithiobacillus caldus* and *A. ferrooxidans*, and demonstrated that this was used as a carbon and energy source by *Sulfobacillus* spp. The results of this study confirmed that glycolic acid in the spent medium of *A. ferrooxidans,* as previously reported by Nancucheo and Johnson [28], was also used, at least in part (~20%), by the *Curtobacterium* isolate (Figure 2).

To mitigate the risk of reactive mineral tailings generating metal-rich, extremely acidic waste drainage waters, they are usually stored under water to limit contact with oxygen. Even so, ferric iron, generated in the aerobic upper layers, can diffuse into tailings and oxidize sulfide minerals in the absence of oxygen [29]. Diaby et al. [8] found that, in pyritic tailings (deposits below the “oxidation front”, the junction between the oxidation and neutralization zones), the dissimilatory reduction of ferric iron was a dominant geochemical process, since ferric iron, produced by iron-oxidizing acidophiles in the aerobic tailings surface and migrating downwards in percolating drainage waters, can act as a terminal electron acceptor, for both heterotrophic and many autotrophic species (including *A. ferrooxidans*), when oxygen is limited or absent. Extremely acidic environments usually contain ferrous and ferric iron in much greater concentrations than those typically found in neutral pH water bodies [30]. The redox potential of the ferrous/ferric couple is relatively high, at pH values less than ~2.0 (~ +680 mV in sulfate-rich liquors; [31]), due to the enhanced solubility of both ionic species of this metal. This makes ferric iron a thermodynamically attractive alternative electron acceptor to oxygen in acidic environments, both for heterotrophic (coupled to organic carbon) and autotrophic (coupled to reduced sulfur or hydrogen) acidophiles [25]. Previously, Nancucheo and Johnson [13] showed that the *C. ammoniigenes* strain IT2 catalysed the dissimilatory reductive dissolution of amorphous ferric hydroxide (concurrent with a corresponding increase in cell numbers), when incubated under anaerobic conditions in cultures containing glucose as the electron donor. This important trait was not previously described for this genus, and adds another species of mesophilic, acidophilic bacteria to the list of those that can use the dissimilatory reduction of ferric iron to support growth in oxygen-limited cultures in highly acidic environments, such as those found in many pyritic mine tailings. Most currently known iron-reducing heterotrophic acidophiles found in pyritic mine tailings are *Proteobacteria* [32]. Interestingly, dissimilatory reduction of ferric iron has also been described for other genera of acidophilic actinobacteria, including *Ferrimicrobium acidiphilum*, *Ferrithrix thermotolerans*, *Aciditerrimonas ferrireducens* and *Acidithrix ferrooxidans* [33], and the novel recently-described genus *Acidiferrimicrobium australe* [11]. In addition, species of acidophilic actinobacteria, except *Aciditerrimonas ferrireducens* and the isolate IT2, also oxidize ferrous iron.

By lowering concentrations of ferric iron, the prime oxidant of sulfide minerals in low pH environments, *C. ammoniigenes* strain IT2 (and other iron-reducing acidophiles) can, in theory, help control the production of metal-rich mine waters, especially where mineral wastes are ecologically engineered to stimulate such bacteria by limiting oxygen ingress and (possibly) promoting the influx of organic electron donors (e.g., algal exudates, [13]). New strategies are required to stabilize the storage of mineral tailings, which represents a long-term engineering and environmental challenge, where, occasionally, catastrophic environmental pollution has occurred due to the failings of the retaining dam of a tailings impoundment [6]. Preventing the oxidation of metallic sulfides in mineral tailings has been highlighted as a key criterion for the ecological restoration of mine tailings by revegetation, and heterotrophic bacteria such as *Curtobacterium* spp. may possibly be used as biological indicators for monitoring mineral tailings during the process of restoration, in order to minimize the solubilization of a variety of transition metals associated with sulfide minerals.

## 4. Conclusions

This study provides further evidence to explain how C. *ammoniigenes*, a moderately acidophilic, heterotrophic actinobacterium, can be found and can proliferate in mine pyritic tailings, where primary producers, such as chemolithotrophic acidophiles like *A*. *ferrooxidans*, sustain the growth of heterotrophic iron-reducing bacteria, which may contribute to mitigating the formation of acidic, metal-rich waters from mineral tailing dumps.

## Figures and Tables

**Table 1:** Comparison of organic substrates utilized by isolate IT2, and *Acidobacterium capsulatum* and *Acidiphilium cryptum* also found in mine tailings. ++, strong growth; +, weak growth; -, no growth. Key: (++) OD_600_ values > 0.6; (+) OD_600_ values between 0.1 and 0.6; (−) OD_600_ < 0.1.

Substrate	IT2	*Acidobacterium capsulatum ^a^*	*Acidiphilium cryptum ^a^*
Glucose	++	++	++
Galactose	++	++	++
Fructose	++	Nd	Nd
Xylose	++	++	++
Mannose	++	++	+
Arabinose	−	++	++
Rhamnose	−	Nd	Nd
Maltose	+	++	+
Lactose	+	++	+
Glycerol	++	−	++
Mannitol	+	−	++
Sorbitol	−	Nd	Nd
Acetic acid	−	Nd	Nd
Citric acid	−	Nd	Nd
Glutamic acid	−	Nd	Nd
Asparagine	−	Nd	Nd
Arginine	−	Nd	Nd
Lysine	−	Nd	Nd
Leucine	−	Nd	Nd
Proline	−	Nd	Nd
Ethanol	−	−	−
Methanol	−	Nd	Nd
Tryptone soy broth	++	Nd	Nd
Yeast extract	++	Nd	Nd

^a^ data from [20]; Nd, not determined.

**Table 2:** Minimum inhibitory concentration (MIC) of some metals (mM) recorded for *C. ammoniigenes* strain IT2, with the highest concentration at which growth of the bacterium was observed indicated in parentheses.

Metal	MIC (mM)
Cu^2+^	15 (10)
Fe^2+^	250 ^a^ (200)
Zn^2+^	125 (100)
Ni^2+^	100 (75)

^a^ The largest concentration tested to avoid precipitation of iron.
